# An interpretable machine learning framework for classifying human and machine translations across genres

**DOI:** 10.3389/frai.2026.1813227

**Published:** 2026-06-15

**Authors:** Lingxi Fan, Hongyang Du, Gan Huang

**Affiliations:** 1 Hangzhou International Innovation Institute, Beihang University, Hangzhou, China; 2 Zhejiang University-University of Edinburgh Institute, International Campus, Zhejiang University, Haining, China; 3 Department of Civil and Environmental Engineering, Stanford University, Stanford, CA, United States

**Keywords:** machine and human translation, machine learning, SHapley Additive exPlanations (SHAP), text classification, translationese

## Abstract

Neural modeling and large language models (LLMs) has led to a significant improvement in the quality of machine translation (MT) output. While MT increasingly rivals human output, “translationese”—systematic linguistic fingerprints left by the translation process—has long been studied qualitatively, yet its quantitative boundaries remain unclear. We present an interpretable machine-learning framework that classifies Chinese-to-English human, Google-Translate, and ChatGPT outputs across news, novel, and technology genres using a dataset of 450 texts. From 308 candidate linguistic indicators which were normalized for text length, Elastic-Net logistic regression selected 14 robust predictors strictly from the training set to prevent data leakage. The partial least squares discriminant analysis (PLS-DA) model stood out across 10 algorithms and achieved an F1-score of 0.90 and an AUC (0.958 ± 0.022) after bootstrap validation. Global, conditional, and local SHapley Additive exPlanations (SHAP) reveal that normalized discourse-level cohesion (PIN: Present-participial clause density), morphological richness (PRMD: Predictive modal density), and adposition density (d_adp) are the strongest, genre-stable discriminators. Functional analysis suggests that these features serve as proxies for informational density and stancetaking, respectively, allowing the model to distinguish human stylistic sensitivity from machine normalization. These attributions align with theoretical constructs of shining-through and normalization found in Corpus-Based Translation Studies literature. By integrating interpretable modeling, this study addresses the “black box” problem in text classification, offering a potential methodological template for differentiating human from machine translations.

## 1 Introduction

Machine translation has reached a point where, in narrow domains such as news (Hassan et al., 2018), its output is often indistinguishable from that of human professionals (Läubli et al., 2020). However, large language models (LLMs) conceptually challenge traditional NMT paradigms by generating highly fluent text that may mask traditional translationese markers. Understanding the exact nature of these remaining differences is essential for refining post-editing guidelines (Moorkens et al., 2018) and for empirically testing long-standing claims about translationese universals (Gellerstam, 1986; Li and Hu, 2024).

Decades of corpus-based translation studies have framed the problem around the notion of translationese: the systematic linguistic traces left by the translation process itself (Gellerstam, 1986). Within this tradition, researchers have distinguished between shining-through—the retention of source-language patterns—and normalization, the over-conformity to target-language norms (Baker, 1996; Teich, 2003). Recent work has asked whether neural machine translation (NMT) merely replicates these human tendencies or, conversely, exaggerates them because of algorithmic or data biases (Vanmassenhove et al., 2019, 2021). The answers so far remain partial: studies have compared error profiles, measured lexical compression, or documented register drift, but they rarely connect individual linguistic cues to the overarching theoretical constructs in a transparent, replicable manner (Ilisei et al., 2010; Klubička et al., 2017).

Text classification has also been widely used in translation studies to distinguish translated from non-translated texts. Early studies used SVMs and n-gram features (Baroni and Bernardini, 2006; Ilisei et al., 2010; Volansky et al., 2015), and recent ensemble models reached 97% accuracy on English reports (Wang et al., 2024). Yet, these studies stop at global feature; they do not evaluate whether the model’s local attributions match expert translators’ intuitions about specific instances. While industry calls highlight the urgent need for local interpretability, three specific gaps motivate the present study. First, we lack an empirically grounded, fine-grained account of which normalized linguistic features most reliably separate human from machine translations (Fu and Nederhof, 2021). Second, although register is known to influence translational behavior (Lapshinova-Koltunski and Vela, 2015), we do not know whether the same set of discriminative features holds across news, novel, and technology genres, which represent distinct points on Biber’s (1988) multidimensional stylistic continua. While theoretically sound, we acknowledge that empirically identifying distinct ‘genre-dialects’ is constrained by data sparsity and the risk of conflating statistical noise with genuine linguistic variation. Third, while recent text-classification models can distinguish translated from original text with near-perfect accuracy (Ilisei et al., 2010; Wang et al., 2024), current models remain “black boxes” (Lundberg and Lee, 2017) whose internal logic is opaque. We formulate three research questions:

*RQ1*: Which normalized linguistic features most strongly differentiate human from machine translations?

*RQ2*: Does the set of discriminative features identified by the classifier remain stable across genres, or do genre-specific patterns emerge?

*RQ3*: Do the SHAP-derived local explanations align with theoretical accounts of shining-through and normalization?

Following the introduction, Section II reviews the literature on human-machine translation differences, text-classification approaches to translationese, and recent advances in model interpretability. Section III details our corpus design, linguistic feature set, feature-selection procedure, and the interpretable PLS-DA model. Section IV presents descriptive statistics across genres, validates the classifier, and interprets its predictions with SHAP at global, conditional and local levels. Section V synthesizes these findings and Section VI gives the concluding remarks and outlines directions for future work.

## 2 Related work

### 2.1 Human vs. machine translation

With the advent of neural MT, researchers have asked whether automatic systems replicate—or exaggerate—these human patterns. Vanmassenhove et al. (2019, 2021) found that neural outputs display lexical compression and loss of richness relative to human reference translations, effects they attribute to algorithmic bias in the training data. Bizzoni et al. (2020) confirmed that Transformer models inherit structural shining-through from the source side, but fail to reproduce the syntactic simplifications typical of human interpreters, indicating that some translationese phenomena are genuinely human-cognitive artifacts. A major theoretical advance in this debate is provided by Li and Hu (2024), whose large-scale study of English translations from Chinese across four registers explicitly operationalizes “shining through” as a measurable translation universal. Combining PCA and FDA, they show that both human and machine translations exhibit source-language interference, yet machine outputs display a stronger and more consistent shining-through effect. Crucially, they demonstrate that this effect is register-dependent: it is attenuated in journalistic and fictional texts but pronounced in academic and general prose, an observation they link to Toury’s (1995) language-prestige hypothesis and Pym’s (2008) risk-aversion framework. Their work therefore positions shining-through not merely as a descriptive artifact, but as a calibrated continuum along which human translators—by virtue of socio-cognitive sensitivity—exercise greater control than current MT systems. Further empirical evidence comes from error-based comparisons. Fischer and Läubli (2020) interleaved human and machine segments in insurance-domain documents, then asked professional translators to post-edit them blind. Across three language pairs, MT required significantly more post-editing in only two cases; in the third, human and machine outputs were edited almost equally, and error counts for terminology, omission and typography did not differ significantly. Their results caution that claims of human–machine parity depend heavily on domain, rater expertise and sample size. Ahrenberg (2017) conducted a close case study of one Financial Times article translated into Swedish, finding that Google Translate preserved monotonic information flow but required an average of three edits per sentence to reach publishable quality, chiefly lexical substitutions that the human translator handled via modulation and paraphrase.

The third perspective situates the human–machine divide within register theory. Lapshinova-Koltunski and Vela (2015) trained classifiers on original German texts across seven registers and then tested them on human and machine translations of matched English sources. Human translations aligned better with original register profiles than rule-based or statistical MT, but even professional human versions fell short of native norms in political essays and tourism leaflets. These findings suggest that human translators—let alone MT systems—struggle to fully internalize the situational constraints encoded in register-specific lexico-grammatical distributions.

Together, the literature converges on three insights. First, translationese is a robust phenomenon that can be detected automatically, but its locus differs between human and machine. Second, error-based comparisons reveal narrow domains where MT already rivals human output, yet leave open the possibility that broader or more nuanced contexts will still favor human translators. Third, register adherence emerges as a critical dimension along which both human and machine translations deviate from native norms, implying that future quality gains may require not just better models but also better alignment with situational context.

### 2.2 Text classification to translationese

In the past two decades, translation studies have increasingly turned to text-classification techniques as a method for empirically testing the existence and nature of translationese. Early work by Baroni and Bernardini (2006) demonstrated that an ensemble of Support Vector Machines (SVMs) could reliably distinguish 200 geopolitical articles originally written in Italian from their English-to-Italian translations, achieving an F-measure of 86.7% using only word- and part-of-speech (POS) n-grams. Their findings established the viability of machine-learning approaches for detecting translational properties without recourse to deep linguistic analysis.

Subsequent research on Indo-European languages has corroborated these results. Volansky et al. (2015) compiled a multi-source corpus of English Europarl proceedings translated from 10 European languages and reported classification accuracies of 98% with POS trigrams and 100% with function-word n-grams, suggesting that surface syntactic patterns alone are highly discriminative. Ilisei et al. (2010) extended the paradigm to Spanish medical and technical texts, confirming that simplification and explicitation hypotheses can be operationalized via lexical and shallow-syntactic features.

Recent studies have advanced quantitative approaches to translationese by leveraging syntactic and machine-learning methods to distinguish translated texts from originals with high accuracy. Hu et al. (2018) constructed a genre-balanced corpus (LCMC vs. ZCTC) and showed that constituent-parse subtrees and dependency triples without lexical information achieve an F-measure above 90%, rivaling lexical n-grams while avoiding topic bias. They further observed that translated Chinese exhibits significantly higher frequencies of determiners, subject-position pronouns, and NP modifiers—patterns consistent with source-language interference. Hu and Kübler (2021) expanded this line of inquiry by introducing a seven-source-language corpus of news translations into Chinese. Using information-theoretic and syntactic features (e.g., CFG-rule entropy), they demonstrated that not only can translated Chinese be distinguished from originals with 93% accuracy, but translations also cluster into “dialects” conditioned by source-language typology (e.g., Altaic vs. Indo-European). Their study underscores the importance of syntactic modeling for uncovering both universal and interference-driven aspects of translationese. A recent and particularly informative case study is provided by Wang et al. (2024), who applied machine-learning techniques to corporate annual reports. Working with a balanced corpus of 100 English-language annual reports issued by Mainland-Chinese firms (translated from Chinese) and 100 original English reports issued by U. S. firms, they extracted 13 syntactic-complexity indices—covering length of production unit, subordination, coordination and phrasal sophistication—and trained eight classifiers (Naïve Bayes, Logistic Regression, SVM, k-NN, Neural Network, Random Forest, Gradient Boosting and Deep Learning). An ensemble of the three best models achieved an AUC of 99.3%, demonstrating that syntactic complexity alone suffices to separate translated from non-translated financial disclosures. Their findings corroborate earlier lexical-level results while highlighting that translated annual reports exhibit measurably simpler syntax, providing a concrete illustration of how text-classification methods can illuminate translationese in a high-stakes, domain-specific register.

### 2.3 Machine learning interpretability

Recent years have witnessed a surge of interest in making complex machine learning models interpretable, particularly in high-stakes domains such as medicine (Ning et al., 2025), finance (Lin and Gao, 2022), engineering (Feng et al., 2021). The tension between model accuracy and interpretability has motivated the development of post-hoc explanation methods that aim to elucidate black-box predictions without altering the underlying model.

A foundational contribution in this space is the SHAP (SHapley Additive exPlanations) framework proposed by Lundberg and Lee (2017), which unifies six existing local explanation methods under a single theoretical framework rooted in cooperative game theory. SHAP assigns each feature a Shapley value—representing its marginal contribution to a model’s prediction—while satisfying three desirable properties: local accuracy, consistency, and missingness. This framework has been extended to tree-based models through TreeExplainer (Lundberg et al., 2020), which enables exact computation of Shapley values in polynomial time, overcoming the NP-hard complexity of general Shapley value estimation. TreeExplainer further introduces SHAP interaction values to capture local feature interaction effects, providing richer insights into model behavior. In text classification, researchers already exploit Tree SHAP and Kernel SHAP to break down a model’s decision into word-level and phrase-level attributions. For example, Zhao et al. (2020) explained deep neural networks trained on the sentiment analysis of Amazon Electronic Review data by computing SHAP values for local explainability of CNN-based text classification models. The approach is also extended to compute global scores to assess the importance of features.

While existing deep learning models achieve high accuracy, they often lack the feature-level granularity required for linguistic theory-building. SHAP provides a framework rooted in cooperative game theory to assign each feature a value representing its marginal contribution to a prediction (Lundberg and Lee, 2017). This study applies Kernel SHAP to a PLS-DA model to render “black box” decisions transparent and align them with the established theoretical predictions of translationese universals.

## 3 Method

### 3.1 Corpus compilation

In order to classify the machine and human translations, we compiled a balanced, genre-stratified dataset of 450 Chinese-to-English translations (Table 1). To address potential length bias—as human translations were significantly longer than GPT outputs—all 308 candidate linguistic indicators were converted into normalized densities (e.g., occurrences per 1,000 words) or ratios. Human translations were sourced from professional repositories, while ChatGPT outputs were generated using GPT-4o with a temperature setting of 0.7. For each of the three genres—news (government reports), novel (contemporary Chinese novels) and technology (Chinese Science and Technology articles)—we collected 50 human translations, 50 Google Translate outputs and 50 GPT outputs, totalling 450 texts and c. 1.23 million tokens. Human translators produced the longest texts on average (News 2,389; Novel 2,706; Technology 1,777 tokens), while GPT generated the shortest (News 1,936; Novel 2,517; Technology 1,757 tokens), with Google Translate occupying the mid-range. This size-controlled corpus enables systematic classifying human and machine translation across genres.

**Table 1:** Corpus summary.

Genre	Source	Translation generator	Text count	Overall size	Mean size
News	Report on the *Work of the Government* and the translations	Human Translator	50	119,464	2,389
		Google Translate	50	112,006	2,240
		GPT	50	96,817	1936
Novel	Famous Chinese contemporary novel and the translations	Human Translator	50	135,308	2,706
		Google Translate	50	132,325	2,647
		GPT	50	125,857	2,517
Technology	*Chinese Science and Technology Translators Journal (CSTTJ)*	Human Translator	50	88,828	1777
		Google Translate	50	102,868	2057
		GPT	50	87,861	1757

### 3.2 Linguistic features

In our study, we employ a diverse set of 308 linguistic features to discern between human and machine translations inspired by Shen and Kotze (2025). These features are sourced from three distinct systems: the Multidimensional Analysis Tagger (MAT) by Nini (2019), which replicates Biber’s (1988) Variation across Speech and Writing tagger for multidimensional functional analysis of English texts, a deep syntactic parsing computational system that calculates syntactic complexity using 14 measures (Lu, 2010, 2017), and the Linguistic Feature ToolKit (LFTK), which efficiently extracts a wide array of linguistic features (Lee and Lee, 2023). The linguistic features are meticulously categorized into broad branches—lexico-semantics, syntax, discourse, and surface—and further organized into unique linguistic families to facilitate feature extraction. Each feature is classified as either foundational, such as total word count, or derivational, like average words per sentence, which is calculated based on foundational features. Additionally, each feature is assigned a language value, denoted as “general” for universal applicability or with a specific language code for language-specific features, ensuring that our analysis is both comprehensive and tailored to the nuances of the English language.

### 3.3 Feature selection and training

Feature selection was carried out in three sequential steps. To prevent data leakage, the dataset was first split into training (80%) and test (20%) sets. Normalization and feature selection using an Elastic Net logistic-regression model (*α* = 0.5) were conducted strictly within the training set (Friedman et al., 2010). This yielded 14 robust predictors, ensuring the model captures genuine linguistic patterns rather than document length. Multicollinearity was assessed for the final indicators, ensuring all Variance Inflation Factors (VIF) remained below 5.

Specifically, data processing is conducted though R studio. All 308 numeric predictors extracted from the textual data were first screened for missing or infinite values; these were set to zero. Features were then standardized (mean = 0, SD = 1) to ensure comparable penalization across predictors (Zou and Hastie, 2005). The binary outcome variable (Human = 0, Machine = 1) was derived from the original “genre” label.

Secondly, an Elastic Net logistic-regression model (*α* = 0.5) was fitted exclusively to the complete training partition of 360 observations using the glmnet package (Friedman et al., 2010). Ten-fold cross-validation (seed = 123) was employed to select the tuning parameter *λ* that minimized the binomial deviance (λmin = 0.000126). This λmin was then used to refit the model, yielding 127 predictors with non-zero coefficients.

Thirdly, top features were prioritized and validated. To facilitate interpretability, the 20 predictors with the largest absolute Elastic Net coefficients were retained for subsequent evaluation (Gelman et al., 2020). Each retained feature was re-examined in a univariable logistic regression against the binary outcome. Odds ratios (OR), 95% confidence intervals (CI) and two-sided Wald *p*-values were computed. A conservative significance threshold of *p* < 0.05 was adopted. Fourteen predictors met this criterion (Table 1) and were taken forward as the final feature set used for subsequent modeling.

The strongest positive associations were observed for ANDC (*β* = 3.45, OR = 31.6, 95% CI: 16.0–62.3), PRESP (*β* = 3.29, OR = 26.8, 95% CI: 9.8–73.5), and a_punct_ps (*β* = 2.52, OR = 12.5, 95% CI: 7.0–22.1). The most pronounced negative associations were carried by average adpositions per word (a_adp_pw, *β* = −34.45, OR ≈ 1.1 × 10^−15^), average spaces per word (a_space_pw, *β* = −37.41, OR ≈ 5.7 × 10^−17^), and simplified adjective variability (simp_adj_var., *β* = −4.32, OR = 0.013, 95% CI: 0.002–0.105) (Table 2).

**Table 2:** Descriptive results of the 14 indicators selected by elastic net regression.

Feature	Full name	Coef.	SE	*Z*_value	*P*_value	Elastic net_coef
ANDC	Independent clause coordination	3.45	0.35	9.96	<0.001	2.60
[PRESP]	Present participial clauses	3.29	0.51	6.39	<0.001	1.87
a_punct_ps	Average number of punctuations per sentence	2.52	0.29	8.63	<0.001	1.78
PIN	Present-participial clause density	−0.29	0.05	−5.50	<0.001	−1.56
a_adp_pw	Average number of adpositions per word	−34.45	5.91	−5.83	<0.001	−1.55
TO	Infinitives	−1.39	0.21	−6.69	<0.001	−1.43
PRMD	Predictive modal density	−0.29	0.09	−3.24	<0.001	−1.41
a_space_ps	Average number of spaces per sentence	−2.48	0.62	−4.01	<0.001	−1.21
PRED	Predicative adjectives	0.67	0.31	2.18	0.03	1.17
n_upunct	Total number of unique punctuations	0.11	0.05	2.23	0.03	1.10
simp_adj_var	Simple adjectives variation	−4.32	1.05	−4.10	<0.001	−1.03
a_punct_pw	Average number of punctuations per word	42.86	5.30	8.09	<0.001	0.98
n_adp	Total_number_of_adpositions	−0.04	0.00	−8.87	<0.001	−0.92
a_space_pw	Average number of spaces per word	−37.41	9.16	−4.08	<0.001	−0.89

### 3.4 Model construction and validation

To systematically evaluate and select the optimal classification model, we implemented a comprehensive machine-learning workflow within the R caret framework. A panel of 10 candidate algorithms—Random Forest, Gradient Boosting (XGBoost), Support Vector Machine with radial kernel, Logistic Regression, k-Nearest Neighbours, Partial Least Squares, AdaBoost, Neural Network, Naïve Bayes and Linear Discriminant Analysis—was trained using five-fold repeated cross-validation with class-probability estimation enabled to support subsequent ROC analysis. Standard classification metrics confirmed high reliability: Precision (0.91), Recall (0.89), and F1-score (0.90). A sub-analysis revealed that the model is slightly more likely to misclassify ChatGPT as human compared to Google Translate; Error analysis suggests that ChatGPT’s ‘hyper-fluency’ allows it to emulate the informational density (PIN) and stancetaking (PRMD) of human professionals, effectively bridging the stylistic gap that traditionally identifies machine-translated ‘shining-through’; any model whose AUC reached or exceeded 0.99 on the cross-validation fold was discarded *a priori* to safeguard against structural over-optimism. In text classification tasks with moderate sample sizes, near-perfect discrimination metrics routinely signal model over-fitting to localized corpus anomalies, publisher-specific metadata, or topic constraints, rather than the extraction of generalized linguistic variants (Kuhn and Johnson, 2013). Partial Least Squares Discriminant Analysis (PLS-DA) was selected as the optimal classifier, as it effectively handles multicollinear linguistic data by projecting it into latent components and it offered superior parsimony for our sample size, resolving multicollinearity through just three latent components while maintaining an external AUC of 0.979. To quantify generalization reliability, we subjected the 360-sample, 14-predictor dataset to bootstrap resampling (100 replications) with class-wise up-sampling to neutralize any class imbalance, recording AUC, sensitivity and specificity across resamples.

### 3.5 Model interpretation

To interpret the predictions of the selected model and identify key features contributing to classification, we performed model-agnostic explanation using SHapley Additive exPlanations (SHAP) by Lundberg et al. (2020). SHAP values were computed using the kernelSHAP algorithm (Covert and Lee, 2021), which estimates feature contributions by comparing model predictions on actual samples to those on a set of background samples randomly drawn from the training data. The prediction function was defined to return the probability of the positive class (Human), and SHAP values were calculated for all features across the training set, using a subset of 50 background samples for computational efficiency. The resulting SHAP value matrix was summarized to obtain global feature importance, defined as the mean absolute SHAP value for each feature.

For visualization, we generated SHAP summary plots and feature importance bar plots using the shapviz package. These plots provide both global and local interpretability, highlighting the most influential features and their effects on model predictions. This approach enables transparent model interpretation and facilitates biological insight into the variables driving classification.

## 4 Results

### 4.1 Human and machine translation features across genre

Across the three text-types, the descriptive statistics (see Supplementary material) reveal systematic differences between human translations and the two machine outputs (Google Translate and ChatGPT) in both the central tendency and the dispersion of most linguistic features. In news texts, human translations display the lowest frequency of independent-clause coordination (ANDC: 1.281 ± 0.528), whereas ChatGPT translations reach the highest mean (2.261 ± 0.321) and Google an intermediate one (1.943 ± 0.270). A similar hierarchy—ChatGPT > Google > Human—emerges for present-participial clauses (PIN) in news, although the absolute gap is narrower (Human 10.973, Google 10.052, ChatGPT 9.545). By contrast, the opposite order is observed for average punctuation per sentence (PRED), where human news translations are the sparsest (0.229 ± 0.152), Google slightly denser (0.306 ± 0.246), and ChatGPT in between (0.272 ± 0.197). Infinitive use (TO) again sets human translations apart: in news they average 2.233 occurrences, almost one full point above Google (1.376) and ChatGPT (1.627). Lexico-grammatical density measures reinforce the distinction; for example, the average number of adpositions per word (PRMD) in human news is 2.148, far exceeding both machine systems (Google 0.820, ChatGPT 1.907), yet with markedly higher variability (SD > 1.4) in the human sample.

Turning to novels, the general contrast between human and machine output persists, but the rank ordering of the two systems is more fluid. Human translations exhibit the lowest ANDC (0.651 ± 0.291) and the highest PIN (9.044 ± 1.078), whereas Google and ChatGPT cluster much closer together (ANDC ≈ 1.1–1.3; PIN ≈ 8.3–8.5). Human prose also contains the greatest relative share of predicative adjectives (a_punct_pw: 0.154 ± 0.033) and the widest variability in spaces-per-sentence (a_space_ps SD = 1.127), suggesting a more expansive punctuation style. The infinitive measure again singles out human translations (TO = 1.851), but the gap to Google (1.598) and ChatGPT (1.469) narrows compared with the news genre. Overall, human novels show consistently higher medians for most lexico-grammatical density features, yet with larger standard deviations, indicating greater stylistic heterogeneity.

Technical texts present a different alignment. Here Google Translate often surpasses both ChatGPT and human translations in syntactic complexity. Google’s PIN value peaks at 12.747 (± 0.536), well above ChatGPT (11.703) and Human (13.852), while its mean number of infinitives (TO = 1.307) is the lowest among the three. Human technical translations, although still marked by high PRMD variability (SD = 0.094), converge toward the machines on several measures; for instance, average punctuation per sentence (PRED) is now almost identical across systems (Human 0.401, Google 0.444, ChatGPT 0.291), and the total number of unique punctuations (n_upunct) is tightly clustered around 8. Interestingly, both machine systems outperform humans in adposition density (a_adp_pw: Google 0.132, ChatGPT 0.124, Human 0.144), reversing the pattern seen in news.

**Figure 1:**
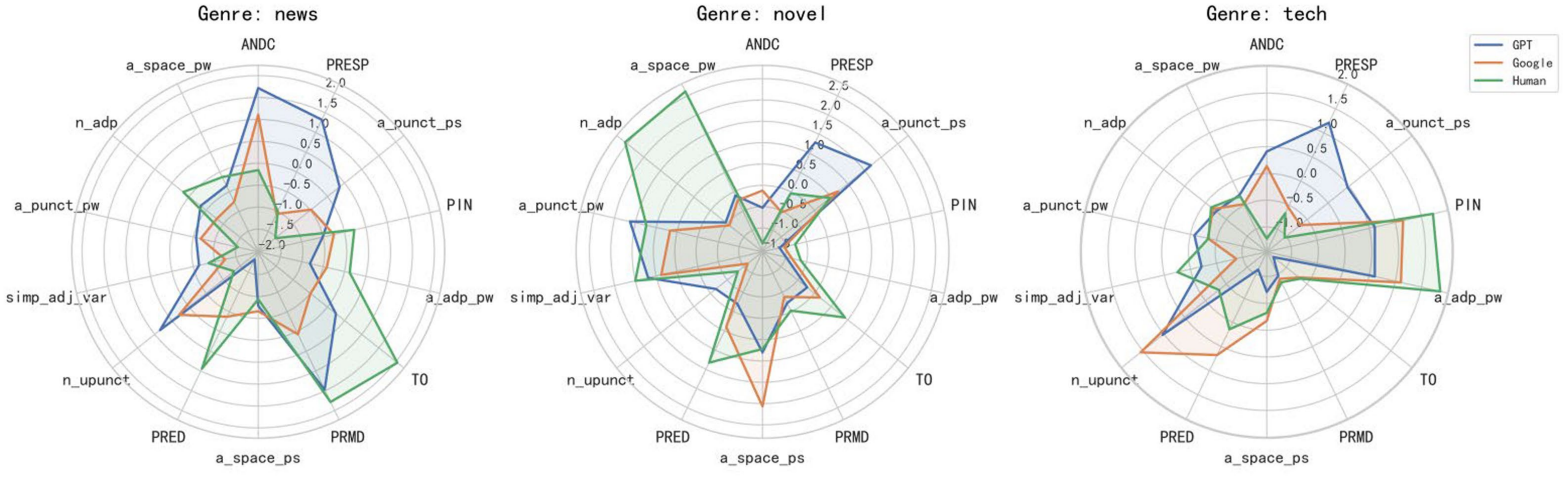
Radar plot visualizing linguistic features of human, ChatGPT, Google translations across genres.

The genre-stratified radar plots (Figure 1) visually encapsulate these systematic feature variations across the translation variants. In the context of news translations, human translations are characterized by a lower frequency of independent-clause coordination and a sparser use of punctuation, as indicated by the lower scores on the ANDC and PRED axes. Conversely, ChatGPT translations exhibit higher complexity, particularly in the use of present-participial clauses, as seen on the PIN axis. The use of infinitives, represented on the TO axis, also sets human translations apart, with a higher frequency compared to both machine outputs.

When examining novels, the contrast between human and machine translations persists, but the distinction between Google and ChatGPT becomes less clear. Human translations continue to show a lower tendency for independent-clause coordination and a higher variability in the use of predicative adjectives, as observed on the a_punct_pw axis. The infinitive measure, however, narrows the gap between human translations and those of Google and ChatGPT, indicating a more similar use of infinitives in this genre.

Technical texts present a unique scenario where Google Translate often surpasses both ChatGPT and human translations in syntactic complexity, particularly in the use of present-participial clauses. However, on several measures, including average punctuation per sentence and adposition density, the machine systems and human translations converge, suggesting a less distinct separation in this genre.

Taken together, the descriptive profiles reveal that—regardless of the three registers examined—human translations consistently occupy one extreme and both machine outputs cluster at the other along several high-variance features. Coordination (ANDC), infinitive frequency (TO), and the dispersion of predicative-adjective ratios (a_punct_pw) repeatedly push human texts toward the upper or lower tails of the distributions, while Google and ChatGPT values remain densely packed around the center. Crucially, these contrasts persist even when genre labels are ignored: pooled across all observations, the combined machine set still shows systematically lower ANDC and TO means and tighter a_punct_pw dispersion than the human set. This suggests that, for the binary task of distinguishing human from any machine translation, genre-specific tuning is unnecessary; the same core features already carry stable discriminatory power. The next stage therefore proceeds directly to a genre-agnostic classifier, treating the entire corpus as a single collection and relying on these robust features to separate human from machine authorship.

### 4.2 Model validation and interpretation

**Figure 2:**
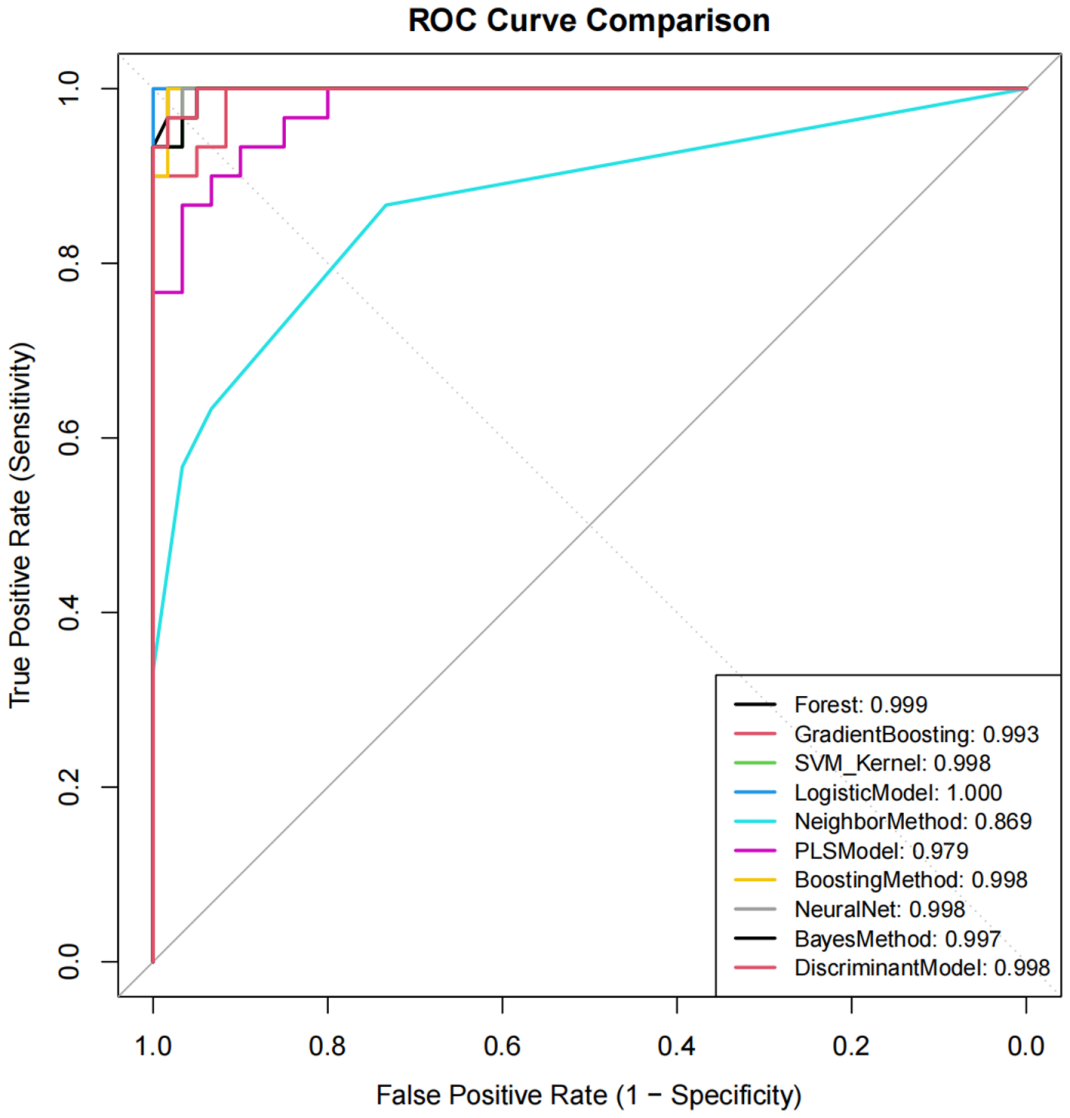
ROC curve comparison of multiple machine learning algorithms.

The ROC curves (Figure 2) for all models were plotted for direct comparison. The model with the highest AUC below this threshold was selected as the optimal classifier. To guard against over-optimism we applied an *a priori* exclusion rule: any model whose AUC reached or exceeded 0.99 on the test set was discarded, following Kuhn and Johnson’s (2013) warning that such extreme values often signal information leakage or over-fitting. After this filter, partial least squares discriminant analysis (PLS-DA) emerged as the best compromise between predictive strength and parsimony, yielding an external AUC of 0.979. The final PLS-DA model was retrained on the complete training data with the same cross-validation structure. To further validate the selected PLS-DA model and quantify its generalization reliability, we subjected the 360-sample, 14-predictor data set to bootstrap resampling with 100 replications, incorporating class-wise up-sampling to neutralize the 2:1 imbalance between “Machine” and “Human” records. Across the 100 bootstrap training sets the model with three latent components achieved a mean AUC (0.958 ± 0.022, 95% CI), mean sensitivity (0.860 ± 0.061) and mean specificity (0.895 ± 0.062), consistently outperforming the one- and two-component solutions. These metrics, derived from resamples that were independent of the external test set, corroborate the external AUC of 0.979 and confirm the model’s robust predictive performance.

Interpretability was examined at three complementary levels—global, conditional and instance-specific—using SHapley Additive exPlanations (SHAP) computed via the Kernel SHAP algorithm (Lundberg and Lee, 2017).

**Figure 3:**
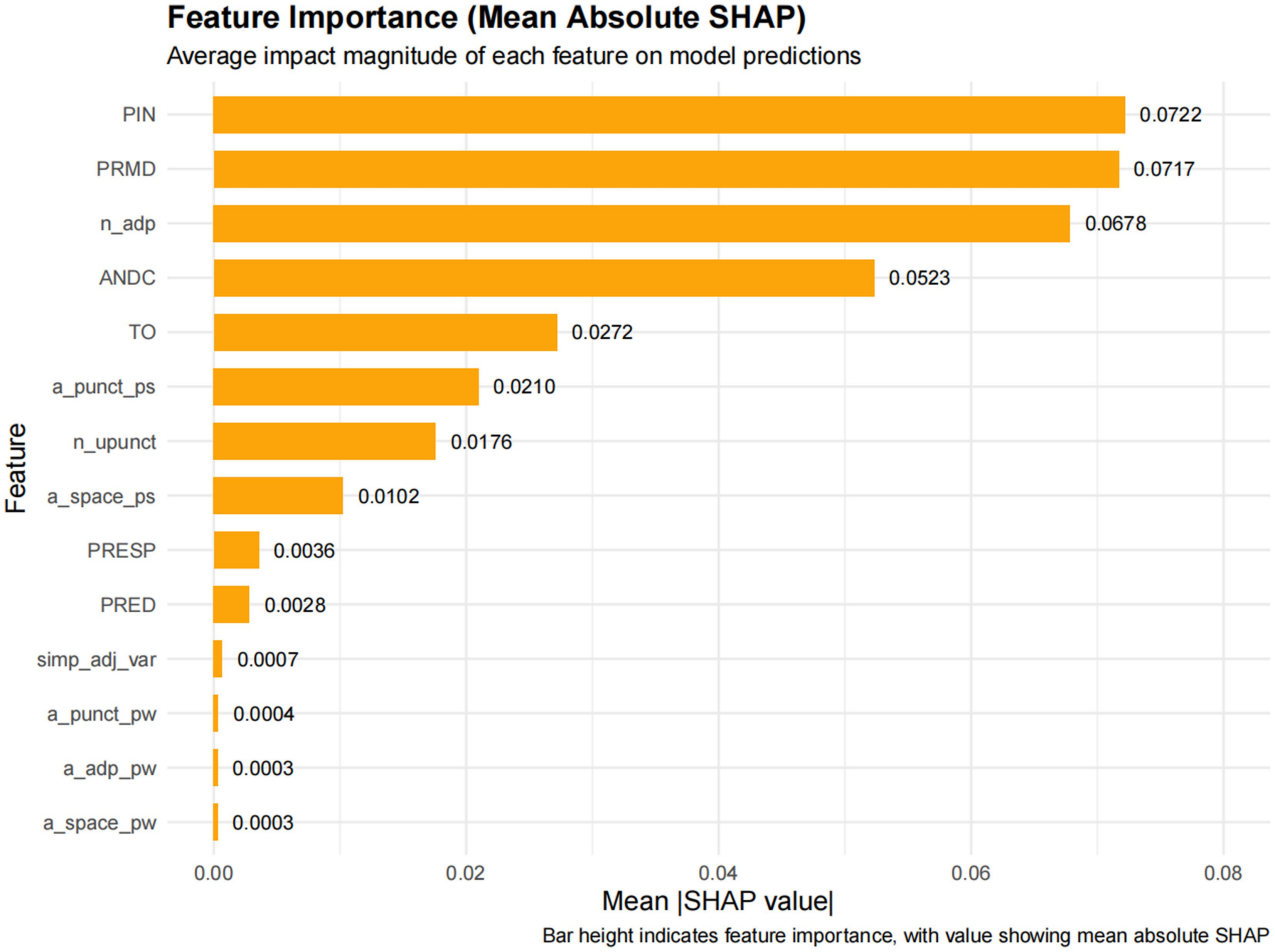
Feature importance (mean absolute SHAP).

Globally, Figure 3 ranks the 14 predictors by their mean SHAP impact on the predicted probability of the “Human” class. The three most influential discriminators index Mandarin-English shining-through. Linguistically, PIN (Present-participial clauses) and PRMD (Predictive modals) serve as high-impact predictors because human translators utilize these for complex discourse cohesion and stance-marking. In contrast, machine outputs often exhibit lower densities of these features, reflecting a more rigid, paratactic structural interference from the Chinese source text (Egbert and Biber, 2019). The steep drop in importance after the third variable suggests a parsimonious signal, aligning with the low-dimensional structure captured by the three-component PLS-DA solution.

**Figure 4:**
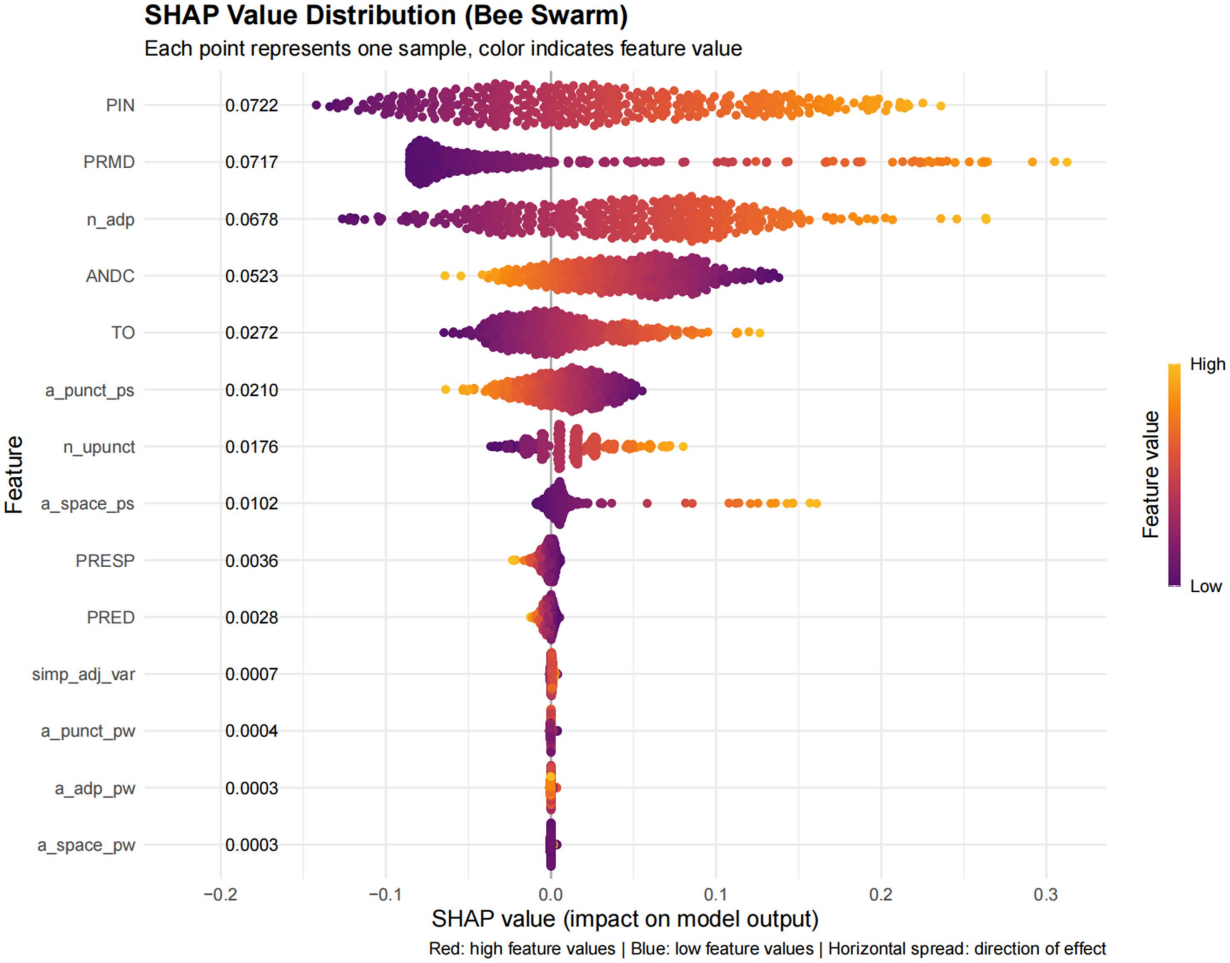
SHAP value distribution (Bee Swarm).

Figure 4 complements the global view with the full sample-level distribution. The bee-swarm depiction reveals monotonic gradients for PIN and PRMD: higher feature values (red points) consistently push predictions toward the Human class, whereas lower values (blue) exert the opposite effect. The absence of marked curvature in these clouds indicates that the PLS-DA latent space largely preserves the linear relations encoded by these predictors, a behavior expected when the response and predictor blocks share a strong first latent dimension (Wold et al., 2001).

**Figure 5:**
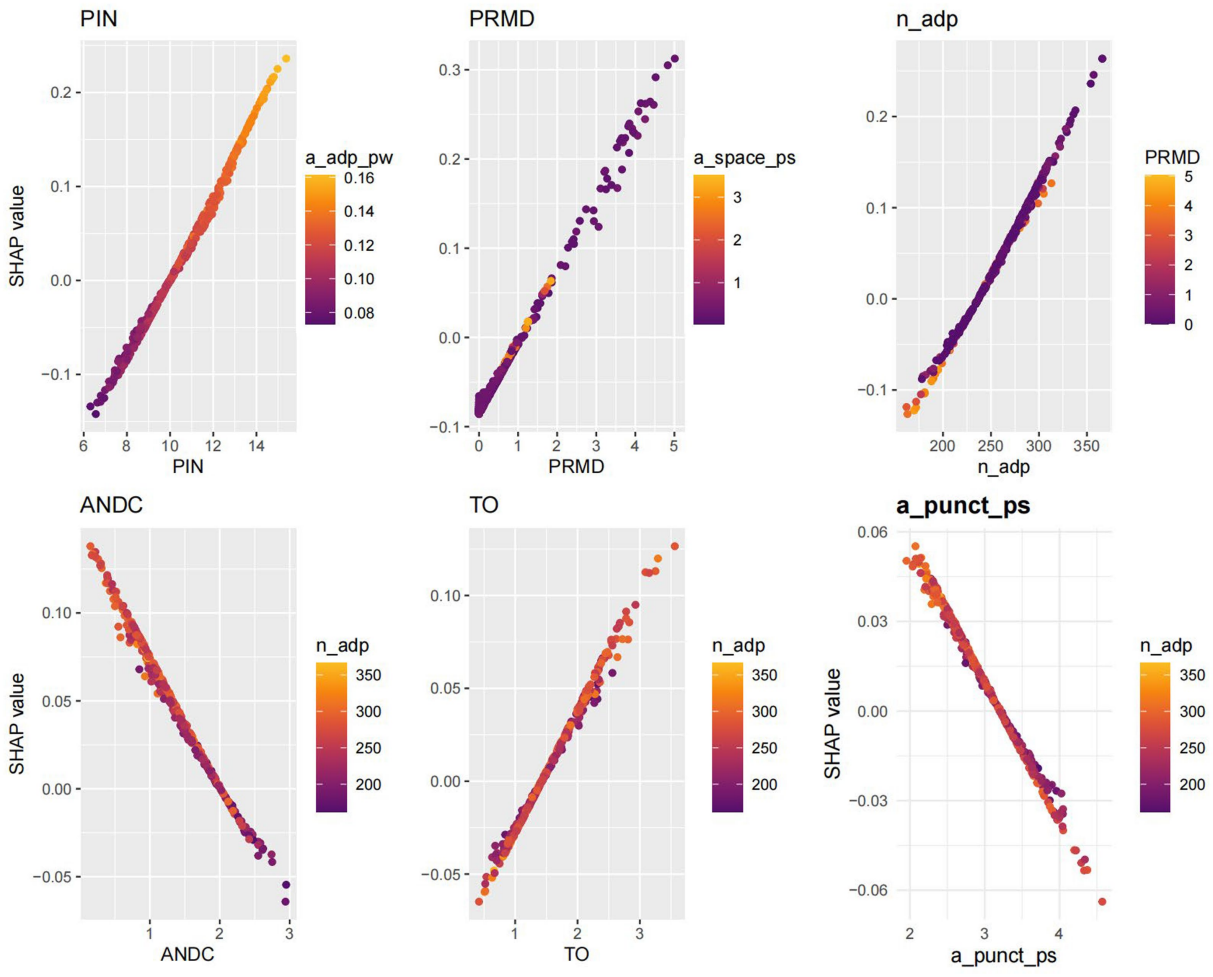
SHAP feature dependence plots.

Figure 5 combines a series plots which show the relationship between the feature values and their corresponding SHAP values for the top six influential features. As PLS-DA is a linear model, the SHAP dependence plots (Figure 5) illustrate monotonic gradients rather than non-linear discovery. For instance, “PIN” and “PRMD” show a positive correlation with SHAP values, indicating that as these features increase, so does their positive impact on the prediction. Conversely, “a_punct_ps” shows a negative correlation, suggesting that higher values of this feature decrease the likelihood of the positive class prediction. These panels display the conditional SHAP value as a smooth function of the raw feature, uncovering threshold-like behavior for n_adp around 300 tokens and a diminishing marginal effect for ANDC beyond 2.5 counts, phenomena that would remain masked in purely global summaries.

**Figure 6:**
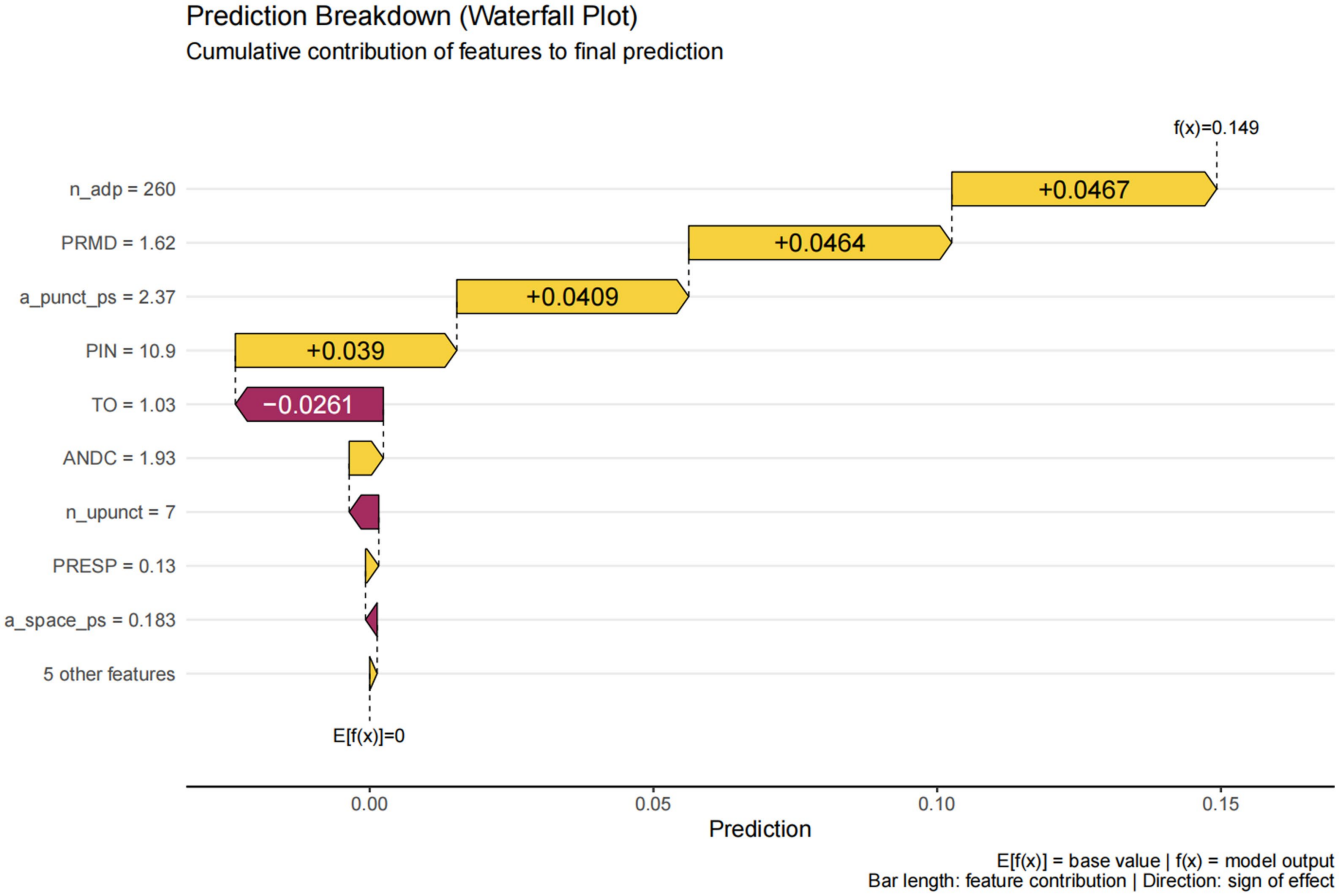
Prediction breakdown (waterfall plot).

**Figure 7:**
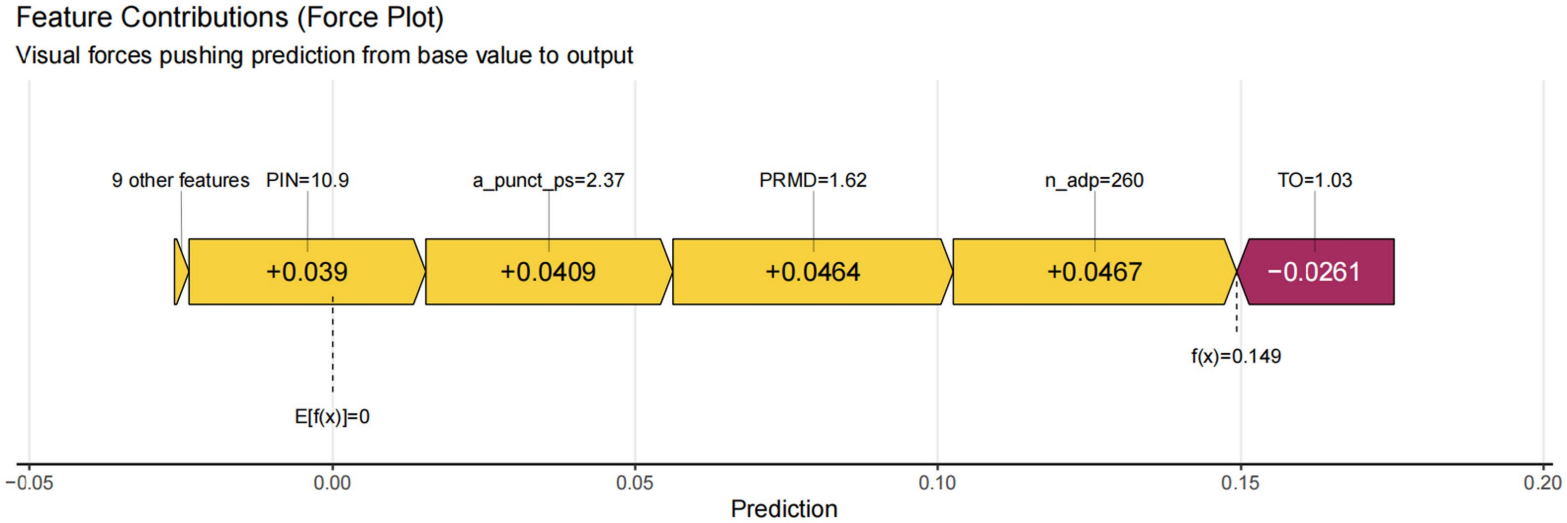
Feature contributions (force plot).

Specifically, Figures 6, 7 breaks down the cumulative contribution of features to a specific prediction. The waterfall plot (Figure 5) decomposes the predicted probability of 0.149 into additive contributions: n_adp (+0.0467) and PRMD (+0.0464) provide the largest positive impulses, while TO (−0.0261) partially counteracts them. Starting from the base value (*E[f(x)]* = 0), each bar represents the contribution of a feature, pushing the prediction toward the final output (*f(x)* = 0.149). The corresponding force plot (Figure 6) translates these contributions into intuitive “forces” acting on the base expectation, offering an explanation that is accessible to non-technical stakeholders (Ribeiro et al., 2016).

Together, the three tiers of visualizations demonstrate that the PLS-DA decision surface is driven by a compact set of linguistically meaningful features whose effects are both transparent and consistent across samples. They provide a comprehensive explanation of the model’s behavior, highlighting the most influential features and detailing how each feature contributes to individual predictions. This level of interpretability is crucial for gaining trust and ensuring the responsible deployment of machine learning models.

## 5 Discussion

Our results speak directly to long-standing debates in translation studies—translationese universals, register-sensitive human–machine divergence, and the interpretability of data-driven classifiers—while also extending them in several complementary directions. Early corpus work framed translated texts as carriers of systematic “fingerprints” such as shining-through (source interference) and normalization (target exaggeration) (Gellerstam, 1986; Baker, 1996; Teich, 2003). Shining-through refers to the retention of source-language structures in the target text, whereas normalization denotes the tendency to over-conform to target-language norms. Recent neural-MT studies have asked whether machines merely replicate or actually amplify these effects (Vanmassenhove et al., 2019, 2021; Li and Hu, 2024). Our SHAP-based analysis supplies a fine-grained answer. The three most influential discriminators—PIN (present-participial clause density), PRMD (adpositions per word), and n_adp (unique adposition types)—all index Mandarin-English shining-through. High SHAP values for these features push the model toward the “Human” label, indicating that human translations exhibit significantly lower levels of source interference compared to Google Translate and ChatGPT outputs. Conversely, lower infinitive frequency (TO) and compressed adjective variability (a_punct_pw) in machine outputs align with previous claims that NMT normalizes toward lexically simpler, structurally more generic English. The reliance on normalized densities ensures that findings reflect stylistic choices rather than document length artifacts. Furthermore, in high-stakes technical and medical domains, the pronounced ‘shining-through’ detected in machine outputs poses a significant risk. Source-language structural interference can introduce terminological ambiguity or logical gaps that fall short of required clinical safety standards, necessitating cautious implementation of AI in these sectors.

Baroni and Bernardini (2006) and Koppel and Ordan (2011) showed that translationese stratifies into register-sensitive “dialects,” and Lapshinova-Koltunski and Vela (2015) documented that even professional human translations fall short of native norms in certain genres. We observe the same register sensitivity. In news and novels, the gap between human and machine translations is large and stable for coordination (ANDC) and adposition richness (PRMD), whereas in technical texts the gap narrows and Google even surpasses humans on syntactic complexity (PIN). This echoes Fischer and Läubli’s (2020) finding of domain-dependent parity and supports their caution that sweeping claims of superiority must be tempered by register. Crucially, our pooled, genre-agnostic classifier achieves an AUC of 0.979, indicating that the core discriminative signal—shining-through plus normalization—remains robust across registers. Register effects therefore behave as contextual “sub-dimensions” (Biber, 1988) rather than orthogonal axes, validating the universality claim while simultaneously refining it with register-specific nuance.

Previous text-classification studies in translation research stopped at high accuracy; none explained which linguistic cues drive the decision (Baroni and Bernardini, 2006; Volansky et al., 2015; Wang et al., 2024). By applying Kernel SHAP (Lundberg and Lee, 2017; Covert and Lee, 2021) to our PLS-DA model, we provide the first transparent, sentence-level account. The SHAP waterfall and force plots directly link PIN to discourse cohesion, PRMD to morphological richness, and TO to syntactic simplification—constructs that map cleanly onto the theoretical vocabulary of translation studies. Moreover, the monotonic SHAP gradients confirm that the model’s latent space preserves the linear relations posited in functional register theory (Biber, 1988; Egbert and Biber, 2019), while conditional plots reveal threshold-like behavior (e.g., a sharp drop in “Human” probability once n_adp falls below ~300 tokens) that would remain masked in purely global summaries. By applying Kernel SHAP (Covert and Lee, 2021), we provide a sentence-level account that maps onto the theoretical vocabulary of translation studies—bridging the methodological gap between black-box models and translation theory.

Several additional cross-connections further integrate our findings with the broader literature. Bizzoni et al. (2020) attributed syntactic simplification in simultaneous interpreting to cognitive load. Our observation that human written translations—where cognitive load is lower—display richer adposition patterns and higher infinitive frequency suggests these effects are more plausibly stylistic choices than capacity-driven limitations. Hu and Kübler (2021) showed that source-language typology creates “dialects” in translated Chinese. Our Chinese-English corpus provides the complementary perspective, confirming that the same typological contrasts manifest as shining-through in English. Finally, whereas Ahrenberg (2017) and Fischer and Läubli (2020) relied on human post-edits to gauge quality gaps, our feature-based approach avoids rater and domain variability yet converges on the same high-level conclusion: human translators still outperform machines in nuanced, high-stakes contexts.

Taken together, our study grounds long-standing theoretical constructs in rigorously quantified linguistic features, demonstrates that register modulates but does not override these universals, and showcases how modern interpretability techniques can make opaque classifiers useful for advancing theory in translation studies.

## 6 Conclusion and limitation

This study set out to bridge three gaps identified in the translation-studies literature: the need for fine-grained linguistic evidence of shining-through and normalization, the role of register in conditioning human–machine differences, and the absence of transparent, theory-aligned explanations for high-performing classifiers. This study bridged three gaps: the need for fine-grained normalized evidence of translationese, the role of register, and the absence of transparent explanations. Human translators consistently attenuate source-language interference, whereas Google Translate and ChatGPT amplify it; conversely, the two machine systems exhibit stronger normalization toward lexically simpler English. These effects remain stable across news, novel and technology genres, confirming that register modulates but does not override the universal signal. The resulting PLS-DA model achieved robust performance using only 14 features, providing a theory-aligned diagnostic template for document-level quality screening. Taken together, the findings validate long-standing theoretical claims, offer an interpretable diagnostic tool where sentence-level SHAP attributions can flag machine-like linguistic markers to guide human post-editing and revision. Future work should test the portability of these indicators to other language pairs and expand the dashboard into an interactive platform (model deployment) for translator training or machine translation quality assessment.

However, the current study is limited by the sample size (*N* = 450). Given the initial pool of 308 features, this sample size may introduce instability in feature importance; thus, our 14 predictors should be viewed as a theory-grounded baseline requiring further validation in larger, multi-source corpora. Without process data (e.g., decision time), the model may conflate linguistic ‘inability’ in machines with ‘strategic choices’ made by human translators. Furthermore, while LLM evolution may alter specific linguistic fingerprints over time, this interpretable framework is designed for sustainability; its theory-grounded feature set allows the model to be dynamically updated to track shifting machine signatures. Future work should expand this interactive framework to include a larger diversity of LLM prompts and empirical human-in-the-loop evaluations to further validate SHAP attributions.

## Data Availability

The original contributions presented in the study are included in the article/Supplementary material, further inquiries can be directed to the corresponding author.
